# Development of a GNSS Buoy for Monitoring Water Surface Elevations in Estuaries and Coastal Areas

**DOI:** 10.3390/s17010172

**Published:** 2017-01-18

**Authors:** Yen-Pin Lin, Ching-Jer Huang, Sheng-Hsueh Chen, Dong-Jiing Doong, Chia Chuen Kao

**Affiliations:** 1 Department of Hydraulic and Ocean Engineering, National Cheng Kung University, No. 1, University Road, Tainan 701, Taiwan; n88021066@mail.ncku.edu.tw (Y.-P.L.); doong@mail.ncku.edu.tw (D.-J.D.); kaoshih@mail.ncku.edu.tw (C.C.K.); 2 Coastal Ocean Monitoring Center, National Cheng Kung University, No. 1, University Road, Tainan 701, Taiwan; sean284@mail.ncku.edu.tw

**Keywords:** GNSS buoy, VBS-RTK positioning, real-time water surface elevations, tides, significant wave height, zero-crossing period, directional wave spectrum

## Abstract

In this work, a Global Navigation Satellite System (GNSS) buoy that utilizes a Virtual Base Station (VBS) combined with the Real-Time Kinematic (RTK) positioning technology was developed to monitor water surface elevations in estuaries and coastal areas. The GNSS buoy includes a buoy hull, a RTK GNSS receiver, data-transmission devices, a data logger, and General Purpose Radio Service (GPRS) modems for transmitting data to the desired land locations. Laboratory and field tests were conducted to test the capability of the buoy and verify the accuracy of the monitored water surface elevations. For the field tests, the GNSS buoy was deployed in the waters of Suao (northeastern part of Taiwan). Tide data obtained from the GNSS buoy were consistent with those obtained from the neighboring tide station. Significant wave heights, zero-crossing periods, and peak wave directions obtained from the GNSS buoy were generally consistent with those obtained from an accelerometer-tilt-compass (ATC) sensor. The field tests demonstrate that the developed GNSS buoy can be used to obtain accurate real-time tide and wave data in estuaries and coastal areas.

## 1. Introduction

Real-time tide data for estuaries are usually employed as boundary conditions in simulations of river water levels to assess flood risks. To study sediment transport in coastal areas, data on tides, waves, and currents are required; however, a simple platform for measuring tide in estuaries and coastal areas is not available. Although a pile can be set up and used as an observation platform, piles are expensive, and the piles in such areas are subject to bed scouring, which increases the difficulty of performing long-term monitoring of tides and waves. Floating data buoys have been verified to be reliable platforms for ocean monitoring and they have been deployed worldwide to provide long-term and real-time meteorological and oceanographic data, such as wind speed and direction, barometric pressure, air and water temperatures, wave height, period and direction, and current speed and direction [1]. However, conventional data buoys do not measure tides or water surface elevations.

Over the past decade, Global Positioning System (GPS) buoys have been developed to measure tide or ocean surface wave data. Kato et al. [2] developed a GPS buoy with a single-frequency Real-Time Kinematic (RTK) GPS receiver to observe tsunamis in real time. The height of the buoy exceeded 13 m, and the weight was approximately 10 tons. Data from the buoy and a land-based GPS receiver were used to determine the position of the buoy using RTK processing software. In their study, the GPS buoy successfully recorded vertical motion in rough and calm seas. Nagai et al. [3] used the GPS buoy developed by Kato et al. [2] to observe tsunamis. The GPS buoy utilized by both Kato et al. [2] and Nagai et al. [3] was deployed within 20 km of the coast. The submerged structure of their buoy has a depth of 8.96 m, which could make its deployment in estuaries and coastal areas difficult.

Harigae et al. [4] utilized a low-cost car-navigation-class GPS receiver and developed a high-pass filter for a buoy to observe waves. Because most GPS positioning errors are below 0.01 Hz and the frequency of ocean waves is approximately 0.1 Hz, a high-pass filter was applied to extract the motion of the GPS-equipped buoy under the excitation of ocean waves, thus minimizing the position error. The accuracies reached several centimeters for the wave height and 5° for the wave direction. However, the system developed by Harigae et al. [4] cannot observe tides. Waseda et al. [5] used the GPS system developed by Harigae et al. [4] on a moored buoy to measure surface waves. Observations made by the buoy were compared with wave records from a nearby observation tower. Both H_1/10_ and T_1/10_ were well correlated with the tower observations, suggesting that their GPS buoy determined the wave heights correctly.

Falck et al. [6] established a real-time reference station for ground motion detection and reference. In their study, tsunamis were observed in real time, and tides were analyzed, however, the focus of this study was on the analysis of tide data and tsunami warnings rather than wave data from the GPS buoy. Doong et al. [7] utilized velocity signals from a GPS receiver on a buoy to obtain wave data. The one-dimensional wave spectrum was derived from the velocity spectrum. The GPS receiver was installed on a moored buoy attached with an accelerometer-tilt-compass (ATC) sensor. Wave data from both sensors were validated. The results indicated that the wave information derived from velocity signals was a reasonable alternative; however, the developed buoy did not measure tides.

Herbers et al. [8] evaluated the capabilities of various GPS-tracked buoys for observing ocean surface waves. Various sensors such as ATC sensor, GPS receiver based on the Doppler shift in GPS signals, and GPS receivers based on the satellite-based augmentation system (SBAS) absolute position data, were mounted on test buoys. The researchers found that the SBAS receivers could accurately resolve horizontal wave orbital displacements. Moreover, when the SBAS receiver was additionally equipped with a precision external antenna, it accurately resolved the vertical displacements. However, the researchers were not concerned with tide measurements.

Kuo et al. [9] utilized GPS buoys along with the precise point positioning (PPP) technique to observe high-frequency sea level variations, which could be identified as waves, meteo-tsunamis, and tides. The PPP technique could be used to overcome limitations of single-point positioning and differential GPS. However, their data were not collected in real-time. Joodaki et al. [10] installed a single GPS receiver on a buoy to measure ocean-surface waves. They applied a simple high-pass filter algorithm to determine the GPS position; however their GPS receiver did not measure tides. Dawidowicz [11] reviewed sea-level-change monitoring systems that use Global Navigation Satellite System (GNSS) technology and suggested that large-scale GNSS buoys could be used to monitor tsunamis, determine tidal levels, calibrate altimeters, and monitor long-term tides. Dawidowicz [11] indicated that the GNSS buoy data of sea surface heights should be verified and noted the difficulty of correcting for the tilt of large-scale GNSS buoys.

The above studies did not measure real-time tides and waves simultaneously using a buoy in estuaries and coastal areas. Because of advances in GNSS technologies, the current accuracy of elevation measurements is on the centimeter level. Hence, developing a real-time GNSS buoy for measuring tides and waves directly is possible. The development of the GNSS buoy will contribute to the advancement of coastal ocean observing systems [12]. This work aims to develop a GNSS buoy that can observe water surface elevations and provide real-time tide and wave data in estuaries and coastal areas without establishing a RTK reference station. The following are the key features of the GNSS buoy developed in the present study:

(1) Virtual Base Station Real-Time Kinematics (VBS-RTK) technology is used for the meteo-oceanographic observations.
(2) The GNSS buoy can receive signals from GPS and global navigation satellite system (GLONASS) and manage L1 and L2 signals.
(3) The GNSS buoy is capable of observing tides and waves simultaneously.
(4) The tidal datum is not influenced by variations in the Earth’s crust.

The accuracy of the monitored water surface elevations from the GNSS-buoy was confirmed by comparing the tide and wave data with those obtained from conventional tide gauge and accelerometer-type data buoys, respectively.

## 2. Methodology

Real-time centimeter-level accuracy positioning based on GPS measurements was developed in the mid-1990s, and is referred to as RTK positioning [13]. One drawback of this technology is that the distance between the rover station and the reference station must not exceed 20 km. The network-RTK was proposed, expanding the baseline limitation to 50 km. According to Wanninger [13], four methods are available for transferring network information to the rover, the VBS being the most widely used. This position technology is called virtual reference station (VRS) RTK, or VBS-RTK.

Other high-accuracy positioning technologies are currently available, such as precise point positioning (PPP) and PPP-RTK. PPP utilizes only a single receiver for positioning. Unlike those of network-RTK, the correction data utilized in PPP are not obtained from the network. They require precise satellite orbits and clocks that may be obtained from organizations such as the International GNSS Service (IGS). However, establishing a reference network for PPP positioning is not necessary.

Mervart et al. [14] combined the advantages of network-RTK and PPP and called it “PPP-RTK”. They assumed that the satellite orbits from the IGS were correct, and utilized data from the ground-based reference network to estimate satellite clock corrections. They also estimated tropospheric delays, receiver clocks and ambiguity parameters. The carrier phase ambiguities were resolved for PPP-RTK clocks corrections, but were not required for PPP clocks corrections. According to Wübbena et al. [15], the accuracy of PPP and PPP-RTK are at the decimeter and centimeter levels, respectively. The decimeter level accuracy may be viewed as a drawback of PPP.

A further drawback of PPP is the long calculation time required for convergence. The integration time for PPP is 30~1800 s, while that for PPP-RTK is 10~50 s. Mervart et al. [14] indicated that the main advantages of PPP-RTK over PPP are faster convergence of solutions and improved kinematic positioning. The main advantages of PPP-RTK over network-RTK are the lower bandwidth requirement caused by less data requiring transmission, and that a 1–2 cm horizontal accuracy can be achieved, even for highly kinematic rovers, at a distance of up to 1000 km from the network.

PPP and PPP-RTK are not suitable for incorporation into GNSS buoys because their convergence times are too long for the real-time monitoring of tides and ocean waves. Furthermore, Taiwan has no service networks for PPP-RTK; hence, the VBS-RTK positioning technology was adopted in this work.

Based on the VBS-RTK positioning technology, this work developed a GNSS buoy for monitoring real-time water surface elevations at the mouths of rivers and in coastal areas. The GNSS buoy contains a buoy hull, data transmission devices, a data logging device, and a RTK GNSS receiver. The methodology for developing a GNSS buoy and the associated theory for obtaining the wave spectrum are discussed in this paper. Section 2.1 describes VBS-RTK positioning technology. To compare the water surface elevations obtained from a RTK GNSS receiver with those obtained from an ATC sensor, the procedures for deriving water surface elevations from the accelerations measured by the ATC sensor are briefly discussed in Section 2.2 and detailed in Appendix A. The procedures for determining the directional wave spectrum and dominant wave direction from the data provided by the GNSS buoy and ATC sensor are provided in Section 2.3 and Section 2.4, respectively, and detailed in Appendix B.

The wave parameters could also be calculated using the raw data obtained from the ATC sensor. An ATC sensor consists of three accelerometers and three magnetometers, and each of these sensors is installed along one of three orthogonal axes. The ATC sensor measures accelerations, inclinations, and the azimuth. Inclinations are computed from the accelerometers and the azimuth is calculated from the magnetometers. The Strapdown Heading Reference manufactured by Watson Industries, Inc. (Eau Claire, WI, USA) was used; detailed specifications are provided in Table 1 [16]. The attitude includes the pitch and roll angles.

### 2.1. VBS-RTK Positioning Technology

In this work, the VBS-RTK positioning technology was adopted to determine the position of the buoy. The VBS-RTK system consists of three components, a GNSS base station network, control center, and rover station. Their functions are as follows:

(1) GNSS base station network: Each base station receives GNSS observation data and transmits raw data to the control center continuously. Currently 78 base stations are located in Taiwan.
(2) Control center: The VBS-RTK control center for positioning computation used in this work is operated by the National Land Surveying and Mapping Center (NLSC), Ministry of the Interior, Taiwan. After 1 September 2014, the NLSC upgraded the network by replacing a GPS system with a GNSS system. Progressive infrastructure via overlaid technology, the commercial software developed by Trimble Navigation, is run in the center. This software includes three modules: Trimble Instrument Configurator, Trimble Ephemeris Download, and Trimble Streaming Manager. Their main functions are as follows:
  - Connecting the control center and each reference station to enable the automatic receipt, storage, and compression of observations from each reference station.
  - The software not terminating the receipt or compression of satellite signals from reference stations during data download.
  - Monitoring the status of the GNSS receiver of each reference station. The GNSS receiver parameters may be configured including the cutoff angle and sampling interval, etc.
  - According to the carrier phase observations, the software calculates continuously the error caused by the multipath, the ionosphere, troposphere, and ephemeris; as well as the integer ambiguity of the carrier phase of L1 and L2.
  - Generating VBS data in Radio Technical Commission for Maritime Services (RTCM) format and transmitting them to the rover station.

When experiments were conducted, the Trimble product GPSNET was running in the control center, and the limit of the baseline length was 30 km. For tropospheric modeling, a modified Hopfield model was utilized to correct for both geometric displacements and tropospheric differences in GPSNET [17]. Kolb et al. [18] at Trimble Terrasat presented a model for extracting characteristic parameters to describe the ionosphere across a network of reference stations using GPS measurements. The main concept was an expansion of the ionospheric delay in terms of a series of orthogonal functions across the area spanned by the piercing points of the ionosphere. In the VRS, the rover did not have to deal with the buildup of these models. Furthermore, Trimble VRS enabled the server to compute the network errors at the rover location from a complex physical model by using information from the full reference station network [19].

The data processing in GPSNET used an optimal Kalman filter to process observations from all reference stations and to model all error sources, including ionospheric and tropospheric effects, orbit and clock errors, multipath interference, and reference station receiver noise. This central processing computed the complete state vector by describing all the aforementioned error sources with an update rate of 1 Hz. The approach involved the Trimble patented factorized multi-carrier ambiguity resolution algorithm to resolve the ambiguity. Furthermore, GPSNET utilized the filtered state vector for the complete network to calculate a virtual reference station dataset at a location near the rover [19].

(3) Rover station: The rover station is a GNSS buoy with a GNSS receiver and a GNSS antenna attached to it.

According to Yeh et al. [20], the procedure of VBS positioning can be summarized as follows:

(1) Pre-process network observations: Establishing the network database and completing coordinate adjustments for each reference station.
(2) Calculating data from regional stations: Collecting continuous observations and the accurate coordinate from each GNSS reference station, thereby establishing the Area Correction Parameters database.
(3) Generating VBS data for the rover: The rover station reports approximate coordinates in National Marine Electronics Association format to the VBS-RTK control center. The VBS-RTK control center calculates the systematic error by interpolation and combines the error with the GNSS observations from the nearby reference station to produce VBS data. Then the VBS data are subsequently transmitted to the rover station in RTCM format.
(4) Calculating the coordinate of the rover station: The rover station receives the VBS data and processes ultra-short-baseline RTK positioning.

In this work, a dual-frequency receiver was installed on the GNSS buoy as the rover station. The receiver receives signals from both GPS and GLONASS. The specifications of the GNSS receiver are listed in Table 2. The circular error probability is 1.2 times the root-mean-square value.

Wu et al. [21] provided detailed information on the technology and precision of the VBS-RTK system operated by the NLSC. They found that for the GPS satellite positioning reference network and VBS-RTK, the total accuracies were approximately 2 cm and 5 cm in the horizontal and vertical directions, respectively. Notably, although the inclusion of a GLONASS into the GNSS network may not improve positioning accuracy, the transmission of accurate data for RTK computation is improved. Because the elevation from the water surface to the location of the GNSS receiver installed on the buoy is known, once the position of the receiver is obtained, the water surface elevation can be easily determined.

### 2.2. Derivation of Water Surface Elevation

An ATC sensor was also installed in the buoy to measure the acceleration, tilt, and azimuth of the moving buoy. The measurements were conducted hourly, each with a duration of 10 min and a sampling rate of 1 Hz. One of the three measurement axes of the accelerometer was perpendicular to the deck of the buoy hull. In this work, the water surface elevations obtained from the GNSS buoy were compared with those obtained from the ATC sensor. Here, we adopted Kao’s method [22] for transforming the ATC accelerations into water surface elevations. The procedures for determining the water surface elevations from the measured accelerations are provided in Appendix A.

### 2.3. Determination of Directional Wave Spectra Using GNSS Data

During the field tests, GNSS data, including the altitude (η) and velocities in the east (u), north (v), and upward (w) directions of the GNSS antenna, was obtained. For analyzing waves, this work assumes that the motion of the GNSS antenna is identical to that of water particles on the free surface. Both *ηuν* and *uνw* data can be used to obtain the directional wave spectrum. In this work, the *ηuν* data were utilized to exploit the available water surface elevations because the altitude (η) is related directly to waves. For simplification, the velocities in the east and north directions are referred to as the x- and y-velocity components, respectively. Appendix B provides detailed information on the method for determining the directional wave spectrum and the peak wave direction from the *ηuν* data provided by the GNSS buoy.

### 2.4. Determination of Directional Wave Spectra Using ATC Data

Equation (B9) in Appendix B is used to calculate the directional wave spectrum from the ATC data. In the equation, the power spectral density (PSD) of the water surface elevation is estimated using the method described in Appendix A. The coefficients in the directional spreading function are calculated based on the formulas provided by Earle [1]. The nearly vertical acceleration, pitch, roll, and azimuth of the ATC sensor were utilized in the analysis.

## 3. Instrumentation, Results and Discussion

A GNSS buoy was established, and laboratory and field tests were conducted to verify the capability of the device and the accuracy of the monitored water surface elevations. Furthermore, the 1-D wave spectrum and the directional wave spectrum of the water surface elevation obtained from the GNSS buoy were compared with those obtained from the ATC sensor. For the following discussion, Taiwan Time is used as the time zone.

### 3.1. Instrumentation

**Figure 1:**
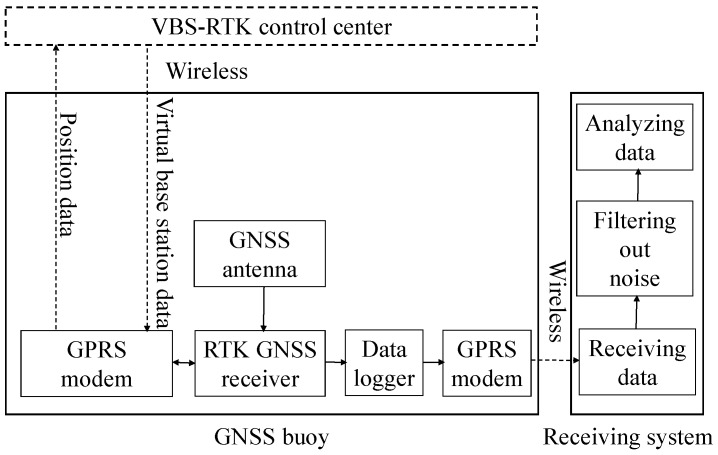
Working principle of a GNSS buoy for monitoring the water surface elevation.

Figure 1 shows the working principle of a GNSS buoy for monitoring the water surface elevation. The GNSS receiver receives signals from satellites and calculates positions. The position data are then transmitted to the VBS-RTK control center by a GPRS modem in a GGA sentence that includes, among other information, the GPS time, latitude, and longitude.

VBS data for RTK positioning are calculated in the control center and transmitted back to the GNSS receiver. Before 1 September 2014, only RTCM ver. 2.3 was transmitted. RTCM ver. 2.3 includes differential GPS corrections, GPS reference station parameters, RTK uncorrected carrier phases, and RTK uncorrected pseudo-ranges. RTCM ver. 3.1 includes GPS extended RTK (L1 and L2), stationary antenna reference points without height information, antenna descriptors, GLONASS extended RTK (L1 and L2), Helmert/Abridged Molodenski transformation parameters, residuals (ellipsoidal grid representation), and receiver and antenna descriptors [23]. In the experiments conducted for this work, the rover station used RTCM ver. 2.3 for RTK positioning. The output rate is 1 Hz. Data, including the altitude above the geoid and the quality index, are transmitted to the receiving system for recording. Using the quality index, we can identify whether the solution type is a phase differential RTK solution with fixed ambiguities, which represent good data and will be used for further analysis.

### 3.2. Laboratory Tests

**Figure 2:**
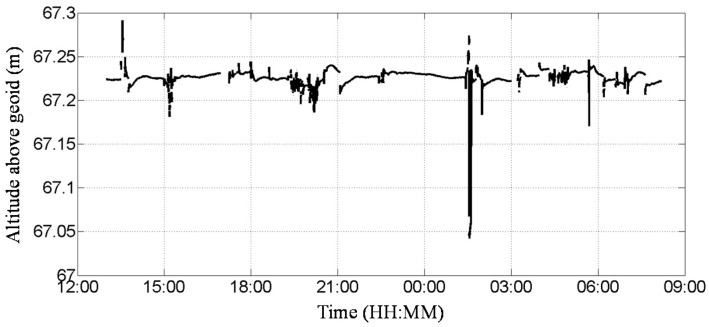
Altitude of the GNSS receiver above the geoid in the static test.

The static and dynamic tests were conducted at the Coastal Ocean Monitoring Center, National Cheng Kung University, Tainan, Taiwan. To ensure a clear sky view, both tests were conducted on the roof of a nearby campus building that has twelve floors. The goal of the static test was to evaluate the accuracy of the monitored altitudes. Time series altitude data received by the GNSS receiver are plotted in Figure 2. An MR-1 receiver with an external PG-A1 antenna (Topcon Positioning Systems, Inc., Livermore, CA, USA) was utilized [24]. We recorded both the data for the altitude above the geoid and the quality index every second, and we chose the altitude above the geoid of the RTK fixed solution for further analysis.

During the period of the static test (24 August, 13:00 to 25 August, 08:00, 2012), a total of 68,400 samples were obtained and 3843 data points without a fixed solution were excluded. The time series of the altitude data are plotted in Figure 2. The obtained average altitude was 67.226 m, the standard deviation was 0.012 m, the maximum was 67.291 m, the minimum was 67.042 m, and the full range was 0.249 m. According to the specifications of the GNSS receiver, the root-mean-square error (RMSE) in the vertical direction was 1.5 cm ± 1.0 ppm (parts per million). The RMSE, which by definition equals the standard deviation (1.2 cm), under the static condition, was smaller than the specified RMSE. Furthermore, the standard deviation of the static data was also smaller than the declared accuracy in the vertical direction of the VBS-RTK (5 cm). There was a drawdown of the altitude data, which was 67.04 m in Figure 2. This might be caused by the multipath effect.

**Figure 3:**
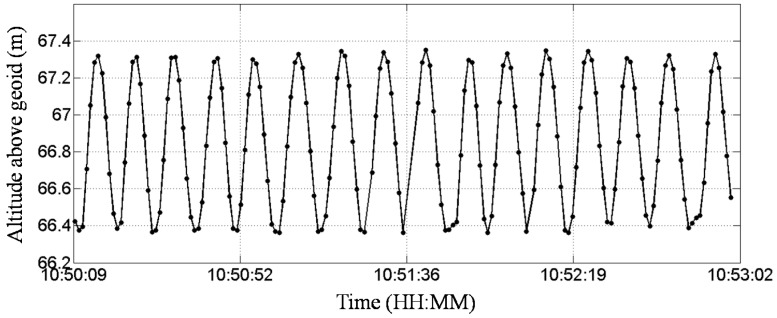
Altitude of the GNSS receiver above the geoid in dynamic test.

The goal of the dynamic test was to check the accuracies of the amplitude and period obtained by a GNSS receiver under a vertical circular motion. The dynamic test was conducted with a well-trained person holding the GNSS antenna and rotating it to simulate a vertical circular motion. During the test, the upside of the GNSS antenna was kept up; the person holding it did not crook his arm, and he kept the rotating speed steady. The solid line with dot symbols in Figure 3 indicates that the GNSS receiver discern the circular motion of the antenna. A ruler was utilized to measure the highest (2.04 m) and lowest (0.9 m) positions of the GNSS antenna. The difference corresponds to a diameter of approximately 1.14 m. According to the data shown in Figure 3, the estimated difference was approximately 0.92 m, which was close to the measured value. In Figure 3, sixteen periods are displayed, the total duration is approximately 3 min, and the average period is therefore approximately 11.2 s. The actual rotation period estimated using a stopwatch was approximately 10.0 s, which is close to the value obtained from Figure 3.

### 3.3. Field Tests

#### 3.3.1. Deployment of the Buoy

**Figure 4:**
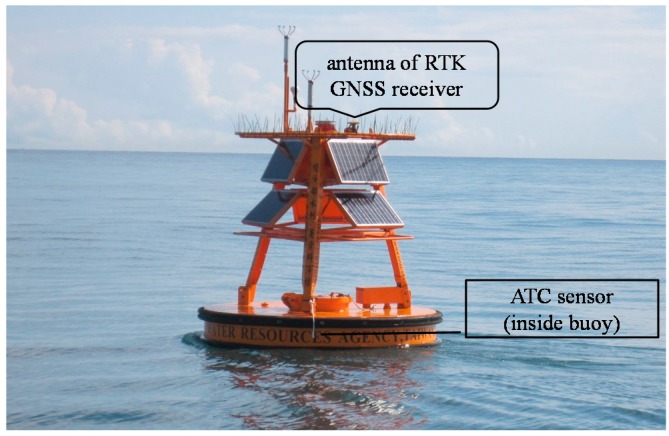
Snapshot of the GNSS buoy deployed in the waters of Suao, Taiwan.

For the field tests, the GNSS buoy was deployed in the waters of Suao (in the northeastern part of Taiwan) from 17 August 2013 to 18 June 2014. The test lasted ten months. Figure 4 shows a snapshot of the deployed buoy. The GNSS buoy is a discus-type with a diameter of 2.5 m. The submerged structure is approximately 1.5 m long. The GNSS buoy was anchored at the seabed with a mooring line longer than the local water depth; therefore, the buoy continued to float and move under the actions of water waves and ocean currents. An ATC sensor was also installed on the buoy to provide wave data for comparison with those obtained from the GNSS receiver.

The buoy was deployed with the assistance of the Coastal Ocean Monitoring Center (COMC), National Cheng Kung University (NCKU), Taiwan. The COMC has been responsible for developing, deploying, and operating the long-term real-time ocean monitoring networks around Taiwan since 1998. To date 17 long-term operational data buoys have been deployed in Taiwan waters for collecting the meteo-oceanographic data at water depths ranging from 10 m to 5600 m. Such sustained, long-term in situ observations will provide monitoring data for numerical ocean model validations [25] and oceanographic research [26].

**Figure 5:**
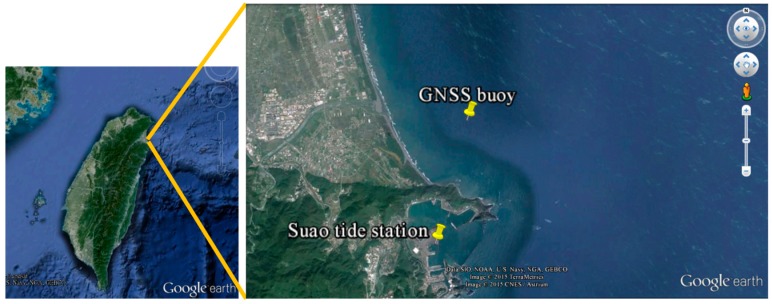
Locations of the GNSS buoy and nearby Suao tide station.

The locations of the GNSS buoy and nearby Suao tide station are shown in Figure 5. The GNSS buoy was located north of Suao Harbor. The local water depth was approximately 20 m, and the nearest distance to the coast was 2.1 km. The Suao tide station is located inside Suao Harbor and run by the Central Weather Bureau (CWB) of Taiwan. Real-time tide data for comparison were acquired from the CWB website. The distance from the GNSS buoy to the Suao tide station was 4 km, and one GNSS base station is located at the Suao tide station.

The standard calibration accuracy of the tide gauge in the Suao tide station, is ±0.025%, the nonlinearity is ±0.02%, and the precision is ±0.01%. The standard range of the tide gauge is 10 m; therefore, the standard calibration accuracy is ±2.5 mm, the nonlinearity is ±2.0 mm, and the precision is ±1.0 mm, respectively.

Based on the working principle illustrated in Figure 1, the GNSS buoy measured the ellipsoidal height, velocities, and quality index hourly. The sampling rate was 1 Hz. The data acquisition system began to acquire samples at the 50th minute of each hour, and the data were collected for 10 min. The monitored data were transmitted via the GPRS modem to the receiving system for further data processing.

#### 3.3.2. Tide Data

**Figure 6:**
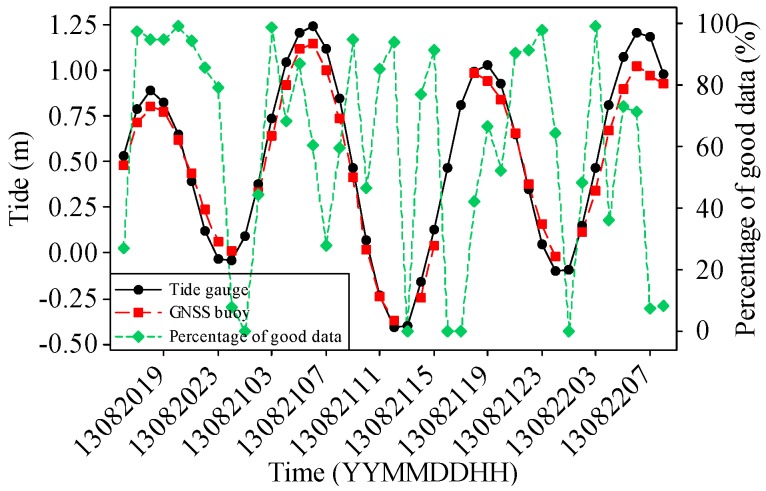
Comparison of tide data obtained from the GNSS buoy and tide gauge (20–22 August 2013).

Figure 6 compares the hourly tide data obtained from the conventional tide gauge with those of the GNSS buoy from 20 August to 22 August 2013. The tide data obtained from the GNSS buoy and the Suao tide station are denoted by the red dashed line with squares and black solid line with circles, respectively. The percentages of good altitude data are plotted as a green dashed line with diamonds. The averaged tide of the GNSS buoy was adjusted to be equivalent to that of the Suao tide station.

Figure 6 shows that the real-time tide data from the GNSS buoy fit well with those of the Suao tide station. The RMSE was 9.5 cm. The RMSE is defined as:

(1) $$\mathrm{RMSE}=\sqrt{\frac{1}{N}\sum_{t=1}^{N}{\left(y_{t}-{\hat{y}}_{t}\right)}^{2}},$$

where $y_{t}$ and ${\hat{y}}_{t}$ represent the tides obtained from the Suao tide station and the GNSS buoy, respectively, and *N* is the number of tide data.

Certain tide data from the GNSS buoy are missing because of the low signal strength of the GPRS at that hour. Notably, four high tides measured by the GNSS buoy were lower than those measured by the Suao tide station. A possible reason is the inclination of the GNSS buoy. Another possible reason is that the GNSS buoy and tide station were at different locations and the tidal waves were affected by the local bathymetry.

#### 3.3.3. Wave Data

For analyzing the wave data, the water surface elevations obtained at two time periods were used. The first period in 2013 was from 21 August, at 02:00 to 22 August, at 17:00, and the second period in 2013 was from 29 August, at 07:00 to 31 August, at 12:00. The data collected during these two periods were selected because the percentages of good altitude data obtained by the GNSS receiver exceeded 95%. During the first period, Typhoon Trami passed through the northern waters of Taiwan, and from 02:00 to 12:00 on 21 August, the typhoon was close to the GNSS buoy.

**Figure 7:**
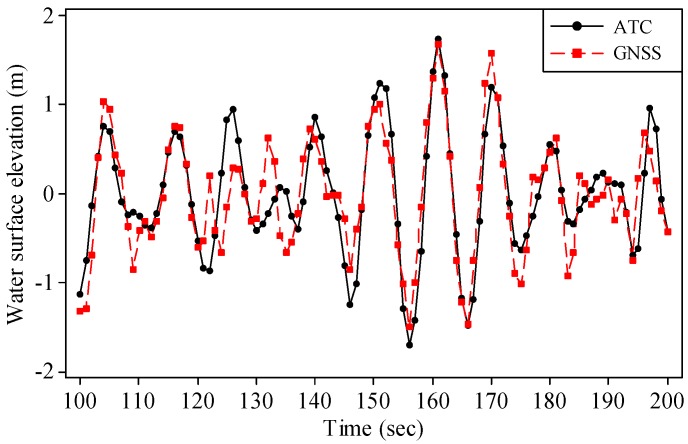
Water surface elevations obtained from the ATC sensor and GNSS receiver at 09:00 on 21 August 2013.

The percentage of good altitude data was 96% at 09:00 on 21 August 2013, and the significant wave height was 2.38 m. Figure 7 compares the water surface elevations obtained at that time from the GNSS receiver with those from the ATC sensor. The black solid line with circles and red dashed line with squares represent the water surface elevations of the ATC sensor and GNSS receiver, respectively. The water surface elevations obtained from the ATC sensor fit well with those from the GNSS receiver, which indicates that the procedures used to obtain water surface elevations from the acceleration measured by the ATC sensor are correct and that both sensors could obtain fairly consistent water surface elevations.

**Figure 8:**
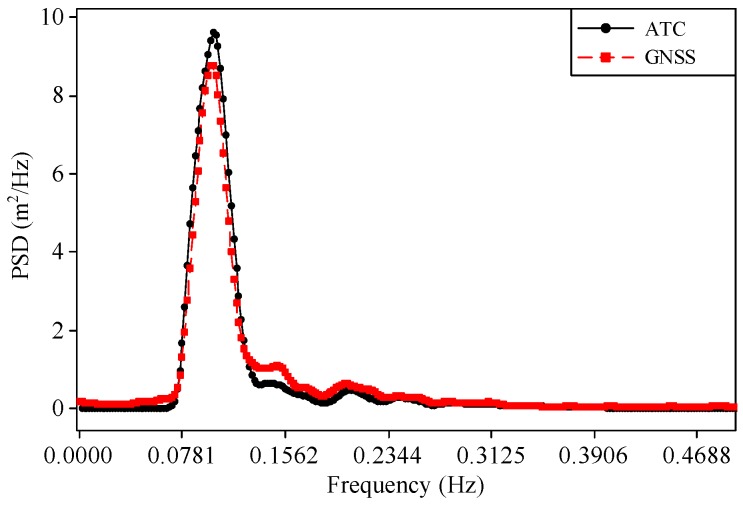
PSD obtained from the ATC sensor and GNSS receiver at 09:00 on 21 August 2013.

A comparison of the one-dimensional wave spectra obtained from the ATC sensor and the GNSS buoy is shown in Figure 8. The vertical axis in Figure 8 is the PSD of the water surface elevations. The one-dimensional wave spectrum and the directional wave spectrum discussed in this section were determined based on the theory presented in Appendix A and Appendix B, respectively. Note that the wave spectra obtained from both sensors are consistent.

The small deviations shown in Figure 7 and Figure 8 may have been caused by the following reasons. Both the water surface elevation and its PSD obtained from the ATC sensor were transformed from the acceleration raw data based on the procedures described in Section 2.2 and Appendix A. The noise filtering and spectral smoothing techniques are empirical. On the other side, the pitch and roll motions of the GNSS buoy may have also caused uncertainty in the measurement of the water surface elevations. In the future, further improvements should be made to account for the effect of the pitch and roll motions of the buoy.

**Figure 9:**
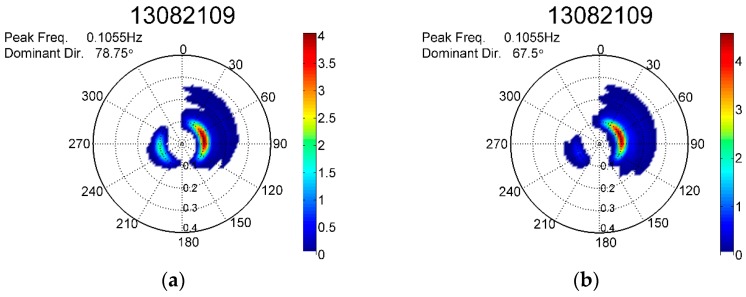
Directional wave spectra obtained at 09:00 on 21 August 2013 from the (**a**) ATC sensor and (**b**) GNSS receiver.

Figure 9a,b shows the directional wave spectra obtained at 09:00 on 21 August 2013 from the ATC sensor and the GNSS receiver, respectively. The peak frequency of the directional wave spectra obtained from both sensors was 0.1055 Hz, and the dominant wave direction from the ATC sensor was 78.75°, and from the GNSS buoy was 67.5°. The peak frequencies were identical, and few differences were observed between the dominant wave directions.

**Figure 10:**
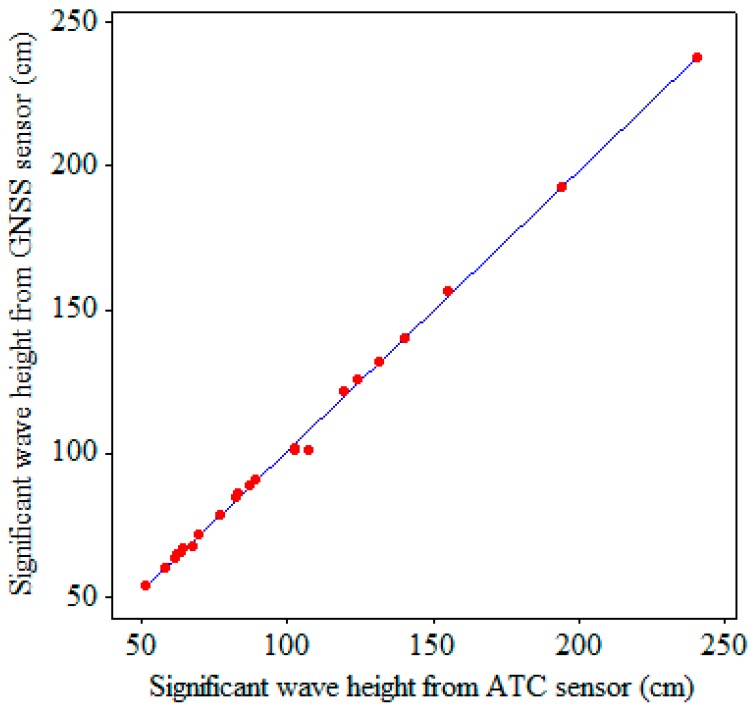
Scatter plot and regression line for the significant wave heights.

**Figure 11:**
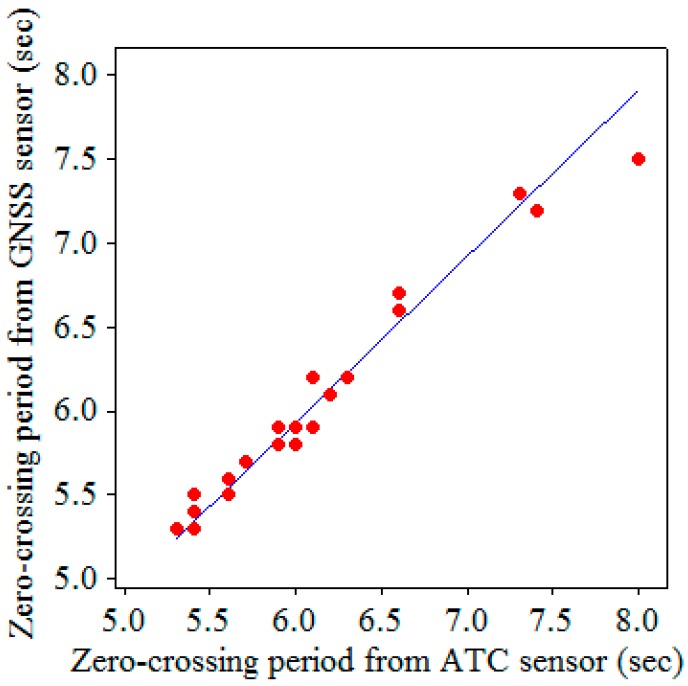
Scatter plot and regression line for the zero-crossing periods.

**Figure 12:**
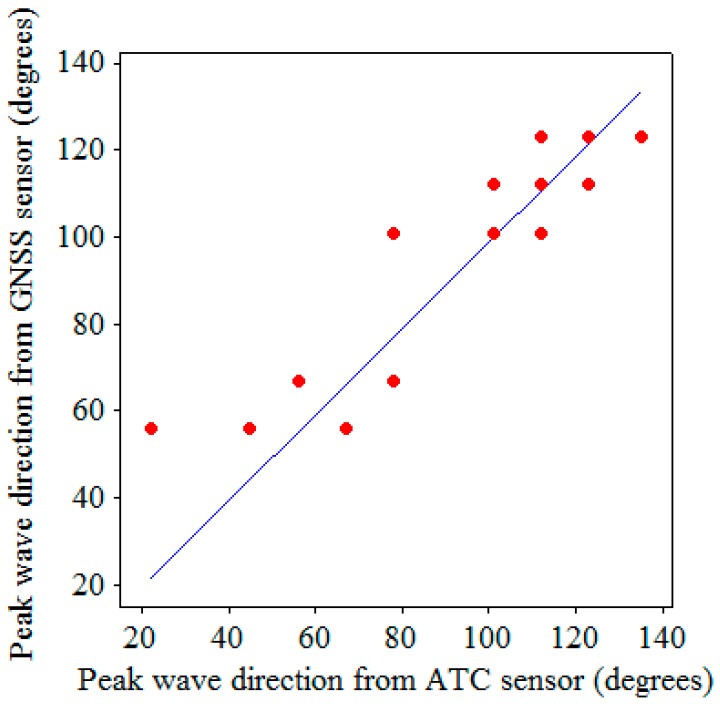
Scatter plot and regression line for the dominant wave directions.

Figure 10, Figure 11 and Figure 12 display the scatter plots and regression lines for the significant wave heights, $H_{S}$, zero-crossing periods, $T_{Z}$, and dominant (peak) wave directions, *DWD*, respectively. The data in these figures represent 23 hourly data, which were chosen from the field test based on the criterion that the percentages of good altitude data exceeded 95% hourly. The numbers of symbols in certain figures were less than 23 because certain data overlapped perfectly. The regressions in Figure 10, Figure 11 and Figure 12 are reasonable because the residuals from the regression lines were random. The linear regression equations for $H_{S}$, $T_{Z}$, and *DWD* are given as follows:

(2) $$H_{S(GNSS)}=0.975H_{S(ATC)}+3.89(\mathrm{cm}),$$

(3) $$T_{Z(GNSS)}=0.989T_{Z(ATC)}(\mathrm{seconds}),$$

and:

(4) $$DWD_{\left(GNSS\right)}=0.99DWD_{\left(ATC\right)}(\mathrm{degrees}).$$

According to these equations, the $H_{S}$, $T_{Z}$, and *DWD* measured by the GNSS receiver are almost the same as those measured by the ATC sensor.

The scatter plot and regression line in Figure 10 show that the significant wave heights obtained from the GNSS buoy were consistent with those obtained from the ATC sensor. The outlier in Figure 11 corresponds to a zero-crossing wave period of 7.5 s from the GNSS buoy and 8.0 s from the ATC sensor. Obviously, the difference is small.

**Figure 13:**
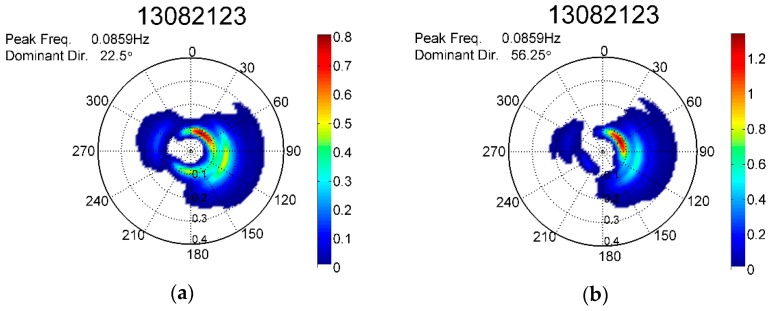
Directional wave spectra obtained at 23:00 on 21 August 2013 from the (**a**) ATC sensor and (**b**) GNSS buoy.

Compared with the scatter plots in Figure 10 and Figure 11, the peak wave directions in Figure 12 are scattered around the regression line. This scattering was caused by the rough resolution of $11.25^{{}^{\circ}}$ ($360^{{}^{\circ}}/32=11.25^{{}^{\circ}}$) in the θ direction. Based on this resolution, a small deviation in both sensors may have caused a direction difference of $22.5^{{}^{\circ}}$ in extreme cases. The outlier in Figure 12 has a maximum direction difference of $33.75^{{}^{\circ}}$. This outlier occurred at 23:00 on 21 August 2013 when Typhoon Trami passed the waters off northern Taiwan. The significant wave heights at that time were approximately the same; at 155 cm from the ATC sensor and 157 cm from the GNSS buoy. A detailed analysis shows that during the period, from 18:00 on 21 August, to 02:00 on 22 August, the peak wave direction experienced large changes. However, the directional wave spectra obtained from the ATC sensor and GNSS buoy at 23:00 on 21 August 2013 were similar, and as shown in Figure 13a,b, the corresponding peak wave directions were $22.50^{{}^{\circ}}$ and $56.25^{{}^{\circ}}$, respectively. Under such circumstances, the peak wave direction was presumably sensitive to the sensors. However, further study is required to clarify this ambiguity.

In the future, wave data obtained under severe sea conditions (e.g., typhoons) will also be collected using both the GNSS receiver and ATC sensor. The differences in the significant wave heights, wave periods, and peak wave directions will be analyzed and compared.

## 4. Conclusions

Based on the VBS-RTK positioning technology, this work has developed a GNSS buoy for monitoring water surface elevations in estuaries and coastal areas. Both laboratory and field tests were performed to test the capability of this buoy. For the field tests, the GNSS buoy was deployed in the waters off Suao (Taiwan) for ten months. The accuracy of the monitored water surface elevations obtained from the GNSS buoy was confirmed by comparing the collected tide and wave data with those obtained from the conventional tide gauge and the accelerometer-tilt-compass (ATC) sensor. The tide levels obtained from the GNSS buoy were consistent with those from a neighboring tide station. The RMSE of the tide data was within 10 cm. The water surface elevations, significant wave heights, zero-crossing periods, one-dimensional wave spectra, directional wave spectra, and peak wave directions derived from the GNSS buoy were consistent with those obtained from the ATC sensor. The field tests demonstrated that the developed GNSS buoy can be utilized as an operational network for providing long-term and real-time tide and wave data in estuaries and coastal areas. Finally, detailed information on the procedures for deriving the water surface elevations from the accelerations measured by an ATC sensor are provided in Appendix A. The procedures for determining the directional wave spectrum and the dominant wave direction from the data provided by a GNSS buoy and an ATC sensor are provided in Appendix B.

## Figures and Tables

**Table 1:** ATC wave sensor specifications.

Item	Parameter	Specification
Acceleration	range	± 1 g
	accuracy	±10 mg
	bias	<±10 mg
	frequency response	20 Hz
Attitude	range	±30°
	accuracy	±0.2° (to 20°), ±0.3°
	frequency response	0.5 Hz
Heading	range	0~360°
	accuracy	±3.0° (magnetic inclination < 75°)
	frequency response	10 Hz

**Table 2:** RTK GNSS receiver accuracy specifications.

Item	Specification
RTK	horizontal: 10 mm + 1.0 ppm (parts per million) × baseline length
	vertical: 15 mm + 1.0 ppm × baseline length
Velocity	0.02 m/s (CEP)

## Appendix A

The step-by-step procedures for determining the water surface elevations from the measured accelerations by an ATC sensor areas follows.

1. Determining the Fourier transform of the acceleration
  The one-sided power spectrum, A(f), the PSD, $S_{a}(f)$, and the phase spectrum, $\varepsilon_{a}(f)$, of acceleration are determined as follows:
  (A1) $${\left|A(f)\right|}^{2}=G_{n}^{2}+H_{n}^{2},$$
  (A2) $$S_{a}(f)=\frac{{\left|A(f)\right|}^{2}}{\Delta f},$$
  and:
  (A3) $$\varepsilon_{a}(f)=\tan^{-1}(\frac{H_{n}}{G_{n}}),$$
  where $\frac{G_{n}}{2}$ and $\frac{H_{n}}{2}$ are the real and imaginary parts of the Fourier transform of the acceleration time series data, respectively, and Δf is the frequency resolution.
2. Calculating the phase spectrum of the water surface elevation
  According to linear wave theory [27], the phase lag between the acceleration of a water particle and the water surface elevation is π. The phase spectrum of the water surface elevation, $\varepsilon_{e}(f)$, is then
  (A4) $$\varepsilon_{e}(f)=\varepsilon_{a}(f)+\pi ,$$
3. Filtering the noise in the acceleration signals
  Because the acceleration signals from the buoys are usually contaminated by noise, Lang [28] proposed an empirical noise correction function for the acceleration obtained by a data buoy. By applying the noise correction function, the noise can be filtered from the PSD of the acceleration. The noise correction function is a linearly decreasing function with respect to frequency and has the following form:
  (A5) $$\mathrm{NC}(f{)}_{LANG}=K\times G[C_{11M}(0.01),C_{11M}(0.02)]\times (F-f)$$
  where K is a constant, G is a function of $C_{11M}(0.01)$ and $C_{11M}(0.02)$ and must be determined, $C_{11M}(0.01)$ and $C_{11M}(0.02)$ are the PSDs of the acceleration at 0.01 and 0.02 Hz, respectively, F is a fixed frequency, and f is the frequency. Lang [28] provided various values of K and F for their discus buoys, which had diameters of 3 m, at different locations. *F* was set to 0.17 Hz for shallow water and 0.15 Hz for deep water. Because the buoy hull and the mooring line used for the present GNSS buoy are slightly different from those used by Lang [28], Kao et al. [22] modified the noise correction function of Lang [28] and suggested the following form for the buoy used in this work:
  (A6) $$\mathrm{NC}(f)=\frac{Max[C_{11M}(<0.03)]}{\left(0.15-f_{MaxC11M}\right)}\times (0.15-f),$$
  where $Max[C_{11M}(<0.03)]$ is the maximum PSD of the acceleration in the frequency range of 0–0.03 Hz, and $f_{MaxC11M}$ is the frequency at which $Max[C_{11M}(<0.03)]$ occurs.
  Accordingly, the modified PSD of the acceleration can be expressed as follows:
  (A7a) $$S_{a}^{\prime }(f)=S_{a}(f)-NC(f),\text{ for }f\geq 0.03\;\mathrm{Hz},$$
  and:
  (A7b) $$S_{a}^{\prime }(f)=0,\text{ for }f<0.03\;\mathrm{Hz},$$
  where $S_{a}(f)$ is the unmodified PSD of the acceleration. Note from Equation (A7b) that signals with frequencies less than 0.03 Hz, which corresponds to a period of 33.3 s, are ignored. Because the periods of typical water waves in the ocean are less than 20 s, ignoring those signals is reasonable.
4. Determining the PSD of the water surface elevations
  According to Hashimoto and Konbune [29], the PSD of the water surface elevations, $S_{e}(f)$, can be determined from that of the acceleration as follows:
  (A8) $$S_{e}(f)=\frac{1}{\omega^{4}}\times S_{a}^{\prime }(f),$$
  where ω is the angular frequency.
5. Smoothing the PSD of the water surface elevations
  Data of 512 points with a time interval of 1 s are used in the fast Fourier transform (FFT). The frequency resolution is approximately 0.001953 Hz before smoothing. According to the spectral smoothing technique described in [30], the PSD of the water surface elevation is smoothed using a Bartlett window of 15 points. The degree of freedom of the PSD is 32. After smoothing, the frequency resolution of the PSD becomes 0.03125 Hz.
6. Computing the wave height and period
  The significant wave height and zero-crossing period are calculated from the PSD of water surface elevations, $S_{e}(f)$, based on the method given by Earle [1].
7. Calculating water surface elevations
  The inverse FFT is used to generate the time-series data of the water surface elevation from the smoothed PSD and phase spectrum of the water surface elevations.

## Appendix B

This appendix presents the methods of determining the directional wave spectrum and the peak wave direction from the *ηuν* data provided by the GNSS buoy, where η denotes the altitude, u and v are the velocities in the east and north directions of the GNSS antenna, respectively. The velocities in the east and north directions are also referred to as the x- and y-velocity components, respectively.

Hashimoto and Konbune [29] described the relationship between the directional spectrum and the cross-power spectrum for any pair of wave properties-such as η, u, and v. Isobe et al. [31] expressed this relationship as follows:

(B1) $$\Phi_{\mathrm{mn}}(f)=\int_{-\pi }^{\pi }I_{m}(f,\theta )I_{n}^{*}(f,\theta )\{\cos[k(x_{mn}\cos\theta +y_{mn}\sin\theta )]\;-i\sin[k(x_{mn}\cos\theta +y_{mn}\sin\theta )]\}S(f,\theta )d\theta$$

where $\Phi_{mn}(f)$ is the cross-power spectrum between the m-th and n-th wave properties, $I_{m}(f,\theta )$ is the transfer function from the water surface elevation to the m-th wave property, θ is the wave propagation angle measured from the x-axis and increases in the counterclockwise direction, the asterisk (*) denotes the conjugate complex, k is the wavenumber, $x_{mn}=x_{n}-x_{m}$, $y_{mn}=y_{n}-y_{m}$, $\left(x_{m},y_{m}\right)$ is the location of the wave probe for the m-th wave property, i is the imaginary unit, and S(f,θ) is the directional wave spectrum.

For the *ηuν* method, the vertical elevation (η) and the velocities in the east and north directions (u and v, respectively) are measured by the same GNSS antenna. Accordingly, $x_{mn}=0$ and $y_{mn}=0$. Thus, Equation (A1) can be simplified as follows:

(B2) $$\Phi_{mn}(f)=\int_{-\pi }^{\pi }I_{m}(f,\theta )I_{n}^{*}(f,\theta )S(f,\theta )d\theta ,$$

with:

(B3) $$I_{m}(f,\theta )=h_{m}(f)\cos^{\alpha }\theta \sin^{\beta }\theta ,$$

where the function $h_{m}$ and parameters *α* and *β* vary with the wave properties and are provided in Hashimoto and Konbune [29].

When Equation (B3) is substituted into Equation (B2), the cross-spectra between the various wave properties can be obtained as follows:

(B4) $$\Phi_{\eta u}(f)=\int_{-\pi }^{\pi }S(f,\theta )h_{u}(f)\cos\theta \;d\theta =C_{\eta u}\left(f\right),$$

(B5) $$\Phi_{\eta v}(f)=\int_{-\pi }^{\pi }S(f,\theta )h_{v}(f)\sin\theta \;d\theta =C_{\eta v}\left(f\right),$$

(B6) $$\Phi_{uu}(f)=\int_{-\pi }^{\pi }S(f,\theta )h_{u}^{2}(f)\cos^{2}\theta \;d\theta =C_{uu}\left(f\right),$$

(B7) $$\Phi_{uv}(f)=\int_{-\pi }^{\pi }S(f,\theta )h_{u}(f)h_{v}(f)\cos\theta \sin\theta \;d\theta =C_{uv}\left(f\right),$$

and:

(B8) $$\Phi_{vv}(f)=\int_{-\pi }^{\pi }S(f,\theta )h_{v}^{2}(f)\sin^{2}\theta \;d\theta =C_{vv}\left(f\right),$$

where $C_{mn}(f)$ is the co-spectrum between the *m*-th and *n*-th wave properties. The directional wave spectrum, S(f,θ), can be expressed as follows:

(B9) $$S(f,\theta )=D(f,\theta )\Phi_{\eta \eta }(f)=D(f,\theta )C_{\eta \eta }(f),$$

where D(f,θ) is the directional spreading function. Note that according to Hashimoto and Konbune [29], both $h_{u}(f)$ and $h_{v}(f)$ are real and the directional wave spectrum is also real. Substituting Equation (B9) into Equation (B4) to Equation (B8), yields the following:

(B10) $$\frac{C_{\eta u}(f)}{C_{\eta \eta }(f)h_{u}(f)}=\int_{-\pi }^{\pi }D(f,\theta )\cos\theta \;d\theta ,$$

(B11) $$\frac{C_{\eta v}(f)}{C_{\eta \eta }(f)h_{v}(f)}=\int_{-\pi }^{\pi }D(f,\theta )\sin\theta \;d\theta ,$$

(B12) $$\frac{C_{uu}(f)}{C_{\eta \eta }(f)h_{u}^{2}(f)}=\int_{-\pi }^{\pi }D(f,\theta )\cos^{2}\theta \;d\theta ,$$

(B13) $$\frac{C_{uv}(f)}{C_{\eta \eta }(f)h_{u}(f)h_{v}(f)}=\int_{-\pi }^{\pi }D(f,\theta )\cos\theta \sin\theta \;d\theta ,$$

and:

(B14) $$\frac{C_{vv}(f)}{C_{\eta \eta }(f)h_{v}^{2}(f)}=\int_{-\pi }^{\pi }D(f,\theta )\sin^{2}\theta \;d\theta ,$$

where:

(B15) $$h_{u}(f)=h_{v}f=2\pi f\frac{\cosh kz}{\sinh kd},$$

The directional spreading function can be expressed as the summation of Fourier series:

(B16) $$D(f,\theta )=\frac{1}{\pi }\{\frac{1}{2}+\sum_{n=1}^{2}[a_{n}^{\prime }(f)\cos n\theta +b_{n}^{\prime }(f)\sin n\theta ]\},$$

The corresponding Fourier coefficients are as follows:

(B17) $$a_{1}^{\prime }(f)=\int_{-\pi }^{\pi }D(f,\theta )\cos\theta \;d\theta ,$$

(B18) $$a_{2}^{\prime }(f)=\int_{-\pi }^{\pi }D(f,\theta )\cos2\theta \;d\theta ,$$

(B19) $$b_{1}^{\prime }(f)=\int_{-\pi }^{\pi }D(f,\theta )\sin\theta \;d\theta ,$$

and:

(B20) $$b_{2}^{\prime }(f)=\int_{-\pi }^{\pi }D(f,\theta )\sin2\theta \;d\theta .$$

Based on linear wave theory, the relationships between the x- and y- components of the velocities and the water surface elevation are as follows:

(B21) $$u=\eta \times 2\pi f\frac{\cosh kz}{\sinh kd}\cos\theta =\eta \times h_{u}(f)\cos\theta ,$$

and:

(B22) $$v=\eta \times 2\pi f\frac{\cosh kz}{\sinh kd}\sin\theta =\eta \times h_{v}(f)\sin\theta ,$$

where d is the still water depth. By applying the Fourier transform, the auto spectra of u and v can be determined as follows:

(B23) $$C_{uu}(f)={\left|F(u)\right|}^{2}={\left|F(\eta \times h_{u}(f)\cos\theta )\right|}^{2}=h_{u}^{2}(f)\cos^{2}\theta \times {\left|F(\eta )\right|}^{2}=h_{u}^{2}(f)\cos^{2}\theta \times C_{\eta \eta }(f),$$

and:

(B24) $$C_{vv}(f)={\left|F(v)\right|}^{2}={\left|F(\eta \times h_{v}(f)\sin\theta )\right|}^{2}=h_{v}^{2}(f)\sin^{2}\theta \times {\left|F(\eta )\right|}^{2}=h_{v}^{2}(f)\sin^{2}\theta \times C_{\eta \eta }(f),$$

where F denotes the Fourier transform.

Combining Equations (B23) and (B24) yields the following:

(B25) $$h_{u}(f)=\sqrt{\frac{C_{uu}(f)+C_{vv}(f)}{C_{\eta \eta }(f)}},$$

Substituting Equations (B17) and (B25) into Equation (B10) yields the following:

(B26) $$a_{1}^{\prime }(f)=\frac{C_{\eta u}(f)}{C_{\eta \eta }(f)h_{u}(f)}=\frac{C_{\eta u}(f)}{\sqrt{C_{\eta \eta }(f)[C_{uu}(f)+C_{vv}(f)]}},$$

Similarly, substituting Equations (B19) and (B25) into Equation (B11) yields the following:

(B27) $$b_{1}^{\prime }(f)=\frac{C_{\eta v}(f)}{\sqrt{C_{\eta \eta }(f)[C_{uu}(f)+C_{vv}(f)]}},$$

Utilizing Equations (B12), (B14), (B15) and (B25), Equation (B18) can be rewritten as follows:

(B28) $$a_{2}^{\prime }(f)=\int_{-\pi }^{\pi }D(f,\theta )[\cos^{2}\theta -\sin^{2}\theta ]d\theta =\frac{C_{uu}(f)-C_{vv}(f)}{C_{uu}(f)+C_{vv}\left(f\right)},$$

Similarly, utilizing Equations (B13), (B15), (B20) and (B25), Equation (B20) can be rewritten as follows:

(B29) $$b_{2}^{\prime }(f)=2\int_{-\pi }^{\pi }D(f,\theta )\sin\theta \cos\theta \;d\theta =\frac{2C_{uv}(f)}{C_{uu}(f)+C_{vv}\left(f\right)},$$

After D(f,θ) is determined, the directional wave spectrum can be calculated using Equation (B9), and the peak wave direction is determined where the maximum PSD occurs.
